# Examination of Factors Influencing SCBA Washing Behavior among Firefighters in Metropolitan

**DOI:** 10.3390/ijerph19042240

**Published:** 2022-02-16

**Authors:** Hyun Sup Park, Seunghon Ham, Jin Hyeok Jeong, Soo Jin Kim, Hyekyung Woo

**Affiliations:** 1 Department of Health Administration, Kongju National University, Gongju-si 32588, Korea; anjv09@smail.kongju.ac.kr; 2 Department of Occupational and Environmental Medicine, Gil Medical Center, Gachon University College of Medicine, Incheon 21565, Korea; shham@gachon.ac.kr; 3 Fire Science Research Center, Seoul Metropolitan Fire Service Academy, Seoul 03312, Korea; 201203014@seoul.go.kr

**Keywords:** firefighter, SCBA (self-contained breathing apparatus), washing, training experience, decontamination behavior

## Abstract

Field-washing decontamination of equipment is an effective way for firefighters to reduce their risk of secondary contamination. No study has yet clarified the factors influencing effective field decontamination of equipment such as self-contained breathing apparatuses (SCBAs). This study sought to examine factors that influence the SCBA washing and decontamination behavior of firefighters. We conducted a questionnaire using the web-based Seoul Metropolitan Electronic Questionnaire System. As of May 2021, the survey had been sent to 3626 of 7198 Seoul career firefighters, and 1940 subjects were selected to participate in the study. Binomial logistic regression and χ2-test analyses were performed. We confirmed that previous training in SCBA washing was an important factor in effective field decontamination of SCBAs. Firefighters should be trained to perform field decontamination procedures systematically and regulations to perform field decontamination before leaving the scene of a fire should be introduced.

## 1. Introduction

Cancer is a leading cause of death among firefighters. Occupational exposure to polycyclic aromatic hydrocarbons (PAHs), organophosphate flame retardants (OPFRs), and polybrominated diphenyl ethers (PBDEs) places firefighters at increased risk for several diseases or cancers [1,2,3,4,5]. While firefighters rely on the use of personal protective equipment (PPE) to respond to fires immediately, their protective clothing often becomes contaminated with toxic chemicals acquired from the fireground [6,7]. Subsequently, off-gassing of these toxins from field-contaminated gear increases the risk of cancer [8,9]. The chronic health effects of greatest concern identified in studies of firefighters were chronic obstructive pulmonary diseases (COPD), heart diseases, and lung cancer. In particular, lung cancer represents the most difficult cancer site to evaluate in epidemiologic studies of firefighters [10].

PPE is essential for firefighters working in hazardous environments [11]. A systematic operating system that includes the use, washing, charging, education, repair and management of PPE not only improves work efficiency, but also enhances the safety and health of firefighters. Among these, the self-contained breathing apparatus (SCBA) is the only equipment that maintains necessary oxygenation of firefighters performing suppression of fires, rescue at fire scenes, including explosions. Therefore, proper use and management of SCBA will affect the health of firefighters [12]. In addition, the proper operation personnel of the SCBA charger to charge the SCBA and the installation environment of the SCBA charging room will affect the charged air quality of SCBA [13]. Therefore, this will also affect the health of firefighters over a long time.

Decontamination of equipment can be achieved by scrubbing with water and dish soap, which can remove PAHs by as much as 85% [14]. Post-fire decontamination interventions in a recent study included routine cleaning of PPE, showering upon return from incidents, and another round of equipment cleaning at the station. Following the interventions, the amount of urinary polycyclic aromatic hydrocarbon metabolites (PAH-OHs) was reduced on average by 40.4% in engineers and by 36.2% in firefighters [15]. Field decontamination has been delineated as an effective way to reduce the risk of exposure to secondary contamination by equipment for firefighters.

Despite the dangers inherent in firefighting, there is currently a lack of information regarding policies for the occupational health and safety of firefighters. Very little remains known about the factors that influence effective field decontamination of firefighters’ PPE, as few studies have focused directly on this topic. One recent study used the Health Belief Model (HBM) to investigate factors affecting decontamination behavior [16]. To the best of our knowledge, however, no study has elucidated factors influencing the field decontamination of SCBAs. Determining the factors that influence firefighters’ field decontamination of SCBAs can help in the design of programs for decontamination training and the establishment of regulations regarding equipment decontamination.

This study examined factors that influence SCBA washing and decontamination behavior among firefighters.

## 2. Materials and Methods

### 2.1. Korean Firefighter’s SCBA Survey (KFSS)

In this questionnaire survey, we focused on the SCBA and the SCBA charger among the PPE and grasped the actual situation. By deriving problems in the fields of human resources, equipment, budgets, and systems through the results of the questionnaire, it was conducted to seek improvements in fire scenes by field.

In the Fire Science Research Center, Seoul Metropolitan, Fire Service Academy, a questionnaire survey was conducted in seven major domains for people in charge of fire suppression and rescue operations working in Seoul metropolitan. The specific contents of the questionnaire survey are as follows;

(1) Storage, use, washing, charging, training, repair, management status and safety management manual recognition of SCBA set (bombe and mask)
(2) Risk awareness of the generation of hazardous substances at the fire scene and the level of occupational exposure
(3) Usage, management of SCBA charger and SCBA charging room installation environment
(4) SCBA bombe inspection and SCBA repair room operation system
(5) Health belief in the use and management of SCBA: Perceived Susceptibility, Perceived severity, Perceived benefits, Perceived barriers, Self-efficacy
(6) Fire incidence and dispatch status
(7) Demographic and sociological information of questionnaire participants

### 2.2. Study Setting

In South Korea, the purchase and distribution of SCBA sets is carried out collectively by the fire department of each province. The central governing body, the National Fire Agency, holds the authority to enact and revise the safety management manual for PPE and the legal provisions for firefighting equipment. The Safety Management Manual and the Fire Equipment Law include information on the use and washing SCBAs, and the installation environment of SCBA charging rooms, but enforcement remains the responsibility of each provincial fire department. Therefore, the personnel in charge, the budget, the system, and detailed operational matters differ depending on the fire department.

### 2.3. Study Design and Subjects

The design of this study is cross-sectional. We conducted a questionnaire using the web-based Seoul Metropolitan Electronic Questionnaire System. As of May 2021, the survey had been sent to 3626 shift workers engaged in fire and rescue operations out of a total of 7198 career firefighters working in the Seoul metropolitan area. Of these, 2031 responded, showing a survey participation rate of 56.0%. The questionnaire was completed via an electronic questionnaire system using a computer or cell phone. First, we sent official text messages to the institutions where survey subjects were employed, and second, we sent e-mails and SMS messages so that the survey participants could actively participate. As a result, at least 30% of respondent fire departments participated in the survey.

### 2.4. Data Collection

**Figure 1:**
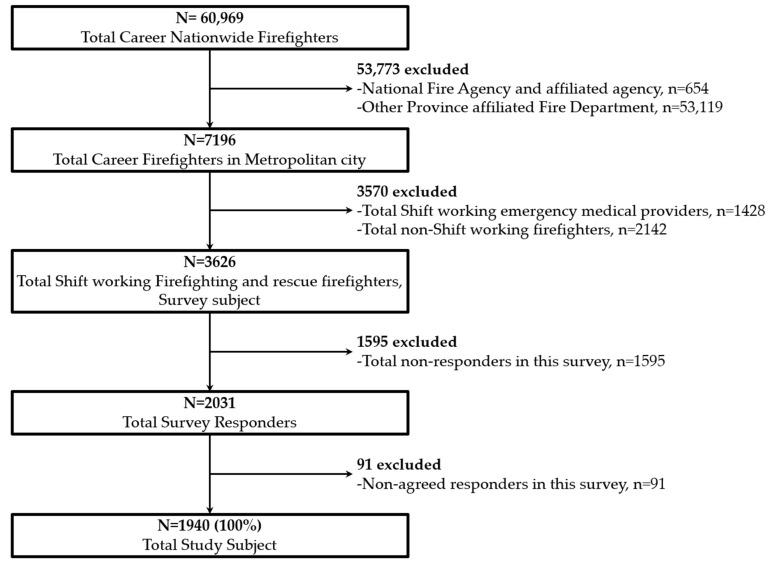
Inclusion criteria.

In this study, firefighters answered questions regarding SCBA washing, training, and information management used at disaster scenes, including fires. To reflect the firefighting scene, we developed questionnaire items through a process of prior research and interviews with experts in the relevant field. Finally, we evaluated the survey items through a pilot survey. The questionnaire was conducted for a total of 19 days from 13 May to 31 May 2021. By explaining the purpose and importance of the questionnaire through official texts, e-mails, and SMS messages, we encouraged the survey participants to enroll. The survey was sent to 3626 shift workers engaged in fire and rescue operations out of a total of 7198 career firefighters working in the Seoul metropolitan area. Of these, 2031 responded, showing a survey participation rate of 56.0%. Of the 2031 survey respondents, only 1940 people who agreed to participate in the study and responded to the survey were analyzed (Figure 1).

The main data collected by the questionnaire survey were demographic and sociological information (sex, age, job ranking, job duties, total years of fire service, total years of shift work in the fire service), fire incidence and dispatch status of participants’ work department (monthly numbers of emergency dispatches in the past year, monthly numbers of dispatches to small or major fires in the past year), and training experience in SCBA use and washing.

### 2.5. Variables

The dependent variable was SCBA decontamination behavior, which was measured by whether participants had engaged in the practice of bombe washing and mask washing over the past year. The independent variables were the following demographic and work-related characteristics: sex, age, job ranking, job duties, total years of fire service, total years of shift work in the fire service, monthly numbers of emergency dispatches in the past year, monthly numbers of dispatches to incipient-stage or free-burning fires in the past year, previous training in SCBA use, and previous training in SCBA washing.

### 2.6. Statistical Analysis

We performed a χ^2^-test analysis to confirm the subjects’ characteristics and associations between the independent variables and decontamination behavior. Binomial logistic regression analysis was performed to confirm factors influencing SCBA washing. All statistical analyses were performed using the IBM SPSS 27.0 program (IBM Corporation: New York, United States).

## 3. Results

### 3.1. Associations between General Characteristics and Decontamination Behavior

Table 1 presents the associations between the general characteristics of participating firefighters and their decontamination of SCBAs. Sex was the only factor that showed a statistically significant difference in bombe washing. However, mask-washing behavior differed significantly for every factor except job duties. Overall, 5.8% of the male firefighters and 18.5% of the female fighters practiced bombe washing, whereas more males (15.9%) than females (3.7%) practiced mask washing. Decontamination behavior was observed most often among participants in their 50s (18.6%). Firefighters ranked as fire captains or above performed mask-washing decontamination (27.3%); those who were responsible for fire suppression practiced mask washing most often, and 20.8% of the firefighters who had worked for 30 years or longer practiced mask washing. Considering total years of shift work in the fire service, firefighters with 10 to more than 20 years performed the most mask washing, at 17.8%. Among firefighters who had been dispatched to a fire emergency 20 times or more per month in the past year, 20.1% practiced mask washing. Similarly, 21.1% of those dispatched to incipient-stage or free-burning fires more than 10 times per month in the past year washed their masks.

### 3.2. Associations between Pre-Training in SCBA and Decontamination Behavior

Table 2 illustrates that both SCBA use and previous training in SCBA washing were associated with decontamination behavior by firefighters. Firefighters who used SCBAs and were trained in SCBA washing were more likely to wash their gear. 

### 3.3. Factors Influencing SCBA Washing

Table 3 presents factors that influenced SCBA washing. The goodness of fit test of the models was statistically significant. The likelihood of practicing bombe washing was 1.86 times higher among firefighters with previous SCBA training (95% CI 1.04–3.31). Female firefighters were 4.66 times more likely to wash their masks compared to their male counterparts (95% CI 2.11–10.32). Firefighters who had received training in SCBA washing were 1.67 times (95% CI 1.15–2.41) more likely to wash their masks. The likelihood of mask washing was 2.06 times higher among firefighters who had been dispatched to 5–10 cases of incipient-stage or free-burning fires in the past month (95% CI 1.02–4.17) and 2.11 times higher (95% CI 1.02–4.38) among those who had been dispatched to 10 or more fire-related incidents, compared to the reference category of not dispatched.

## 4. Discussion

SCBAs is one of the personal protective equipment devices that supplies oxygen to firefighters at all fire scenes. To promote the occupational health of firefighters, various factors related to SCBAs should be considered [17,18]. Washing the SCBA prior to entering the fire department means reducing the occupational exposure of firefighters by primarily removing pollutant-contaminated materials from the scene of a fire. This can be seen as an emergency decontamination process stipulated in the National Fire Protection Association (NFPA) code 1851 [19], and is extremely important in terms of risk removal for occupational health of firefighters. Therefore, SCBA emergency decontamination at the fire scene must be performed. In our study, the probability of SCBA washing behavior at fire scenes was higher in the group with educational experience. This explains the reason behind the SCBA cleaning method, its importance and purpose and thus should be included for firefighters in present and future. In addition, SCBA washing should be included not only at the fire scene but also after compartment fire behavior training (CFBT) conducted at the fire academy, given that CFBT exposes firefighters to pollutants [20].

According to a survey study on the use and management of turnout gear among metropolitan firefighters in South Korea, 9.1% answered that they had removed their turnout gear before boarding a fire fighting vehicle at the scene of a fire after completing firefighting activities. In addition, 41.6% and 48.3% of the respondents answered that they removed their turnout gear in the fire fighting vehicle and garage of the fire department [21]. This means that only a small number of firefighters in South Korea are carrying out decontamination at the fire scene. According to the NFPA Code, emergency decontamination procedures for personal protective equipment at fire scenes are legally stipulated [19,22]. In South Korea, the disaster scene standard operation procedure (SOP) 108 stipulates the return procedure for the fire activation team, but does not include on-scene decontamination. These legal regulations and safety culture would have influenced Korean firefighters to carry out decontamination actions at fire scenes. Therefore, we believe that Korea’s SOP 108 revision and education it is based upon are necessary in the future.

In this study, we first explored the SCBAs decontamination behavior of firefighters and confirmed previous training in SCBA washing to be a key factor. Firefighters who used SCBAs and were trained in SCBA washing were more likely to wash their gear, and pre-training in SCBA washing was an influencing factor while pre-training in SCBA use was not. These findings imply that pre-training in SCBA washing is more important than pre-training in SCBA use regarding SCBA washing behavior. Based on the study sample group, female firefighters were more likely to practice bombe washing than their male counterparts. Firefighters dispatched to five or more insipient-stage or free-burning fires per month in the past year were more likely to have washed their masks than those who had not been dispatched to a fire. While our study observed that sex influenced the practice of bombe washing, the proportion of female firefighters was far smaller than that of male firefighters at only 2.8%. Future studies should use robust evidence to investigate whether sex represents a significant factor influencing SCBA washing behavior. The results of this study imply a possible need for intervention to increase SCBA washing among firefighters.

A previous study reported that the constructs of the Health Belief Model influenced the individual safety behavior of farmers [23]. Among theoretical constructs, norms and self-efficacy were reported to increase the prediction of current decontamination behavior by firefighters [16]. However, to date, no study has explored multiple demographic and work-related characteristics of firefighters, and their impact on the practice of washing SCBAs. The results of the present study indicate that firefighters who already have experience in SCBAs washing bear an increased awareness of the importance of decontamination. Another implication of this study is that the introduction of field decontamination training programs may provide an avenue to an improved occupational health policy that better serves firefighters. Such training and educational programs can facilitate behavioral changes and lead to firefighters engaging in decontamination of their equipment [24].

The main limitation of this study is a lack of generalizability. The study used firefighter data from only the city of Seoul, in Korea. Research conducted elsewhere using the tools of this study may produce different results. In addition, due to the cross-sectional nature of the study, the time of investigation may have skewed the study results. In future studies, it is necessary to investigate and gather data concerning contamination during the use of SCBA [25].

The significance of this study is that, to the best of our knowledge, it is the first to propose training in field decontamination for the occupational health of firefighters. The findings show the necessity of training based on the collection of firefighters’ demographic information and work history at a national level. This study serves as an example of occupational health research among firefighters.

## 5. Conclusions

This study confirmed that previous training in SCBA washing is an important factor in decontamination of SCBAs while previous training in SCBA use is not. When distributing firefighting equipment, it is necessary to train personnel in field decontamination according to standard operating procedures. Moreover, it will be necessary to introduce regulations to perform field-washing procedures prior to returning from the scene of fire-related incidents. The large-scale Firefighter’s SCBA Survey could promote firefighters’ health with greater accuracy. Currently, there is no regular large-scale survey related to the job of firefighters except for mental health surveys. In the future, it will be necessary to investigate the work environment in consideration of the occupational characteristics of firefighters.

## Figures and Tables

**Table 1:** Associations between general characteristics and decontamination behavior.

*n* = 1940										
Characteristics	Total		Bombe Washing (Yes)				Mask Washing (Yes)			
	*n*(%)		*n*(%)		χ^2^	*p*-Value	*n*(%)		χ^2^	*p*-Value
**Sex**					†	<0.001			5.948	<0.015
Male	1886	(97.2)	110	(5.8)			300	(15.9)		
Female	54	(2.8)	10	(18.5)			2	(3.7)		
**Age**					3.686	<0.297			11.410	<0.010
20–29	87	(4.5)	9	(10.3)			9	(10.3)		
30–39	480	(24.7)	32	(6.7)			60	(12.5)		
40–49	538	(27.7)	28	(5.2)			78	(14.5)		
50≤	835	(43.0)	51	(6.1)			155	(18.6)		
**Job ranking**					4.871	<0.301			15.750	<0.003
Firefighter	228	(11.8)	17	(7.5)			22	(9.6)		
Senior firefighter	310	(16.0)	22	(4.7)			40	(12.9)		
Fire sergeant	362	(18.7)	17	(4.7)			60	(16.6)		
Fire lieutenant	974	(50.2)	57	(5.9)			162	(16.6)		
Fire captain≤	66	(3.4)	7	(10.6)			18	(27.3)		
**Job duties**					4.382	<0.112			1.906	<0.386
Fire suppression	1176	(60.6)	65	(5.5)			189	(16.1)		
Rescuer	288	(14.8)	16	(5.6)			37	(12.8)		
Others	476	(24.8)	39	(8.2)			76	(16.0)		
**Total years of fire service**					5.131	<0.274			13.946	<0.007
<1	97	(5.0)	10	(10.3)			11	(11.3)		
1–10	477	(24.6)	33	(6.9)			54	(11.3)		
10–20	429	(22.1)	20	(4.7)			67	(15.6)		
20–30	740	(38.1)	44	(5.9)			129	(17.4)		
30≤	197	(10.2)	13	(6.6)			41	(20.8)		
**Total years of shift work in the fire service**					4.769	<0.190			9.550	<0.023
<1	121	(6.2)	13	(10.7)			12	(9.9)		
1–10	644	(33.2)	38	(5.9)			84	(13.0)		
10–20	569	(29.3)	35	(6.2)			101	(17.8)		
20≤	606	(31.2)	34	(5.6)			105	(17.3)		
**Monthly number of emergency dispatches in the past year**					0.666	<0.717			13.328	<0.001
1–10	625	(32.2)	39	(6.2)			85	(13.6)		
10–20	723	(37.3)	41	(5.7)			98	(13.6)		
20≤	592	(30.5)	40	(6.8)			119	(20.1)		
**Monthly number of dispatches to incipient-stage or free-burning in the past year**					2.783	<0.429			22.959	<0.0001
None	118	(6.1)	7	(5.9)			11	(9.3)		
1–5	990	(51.0)	53	(5.4)			125	(12.6)		
5–10	444	(22.9)	33	(7.4)			84	(18.9)		
10≤	388	(20.0)	27	(7.0)			82	(21.1)		

SCBA: self-contained breathing apparatus. † Fisher’s exact test.

**Table 2:** Associations between pre-training in SCBA and decontamination behavior.

*n* = 1940										
Characteristics	Total		Bombe Washing				Mask Washing			
	*n* (%)		*n* (%)		χ^2^	*p*-Value	*n*(%)		χ^2^	*p*-Value
**Pre-training in SCBA use**					4.466	<0.035			10.803	<0.001
Yes	1198	(61.8)	85	(7.1)			212	(17.7)		
No	742	(38.2)	35	(4.7)			90	(12.1)		
**Pre-training in SCBA washing**					9.223	<0.002			21.159	<0.0001
Yes	1017	(52.4)	79	(7.8)			195	(19.2)		
No	923	(47.6)	41	(4.4)			107	(11.6)		

SCBA: self-contained breathing apparatus.

**Table 3:** Factors influencing SCBA washing.

*n* = 1940				
Potential Risk Factors	SCBA Washing Behavior			
	Bombe		Mask	
	AOR (95%CI)		AOR (95%CI)	
**Sex**				
Male	1.00		1.00	
Female	4.66	(2.11–10.32)	0.26	(0.06–1.12)
**Age**				
20–29	1.00		1.00	
30–39	0.77	(0.28–2.07)	0.92	(0.37–2.27)
40–49	0.64	(0.19–2.16)	0.80	(0.29–2.20)
50≤	0.62	(0.15–2.63)	0.96	(0.31–3.01)
**Job ranking**				
Fire captain≤	1.00		1.00	
Firefighter	0.17	(0.03–0.81)	0.49	(0.14–1.69)
Senior firefighter	0.31	(0.08–1.28)	0.87	(0.30–2.54)
Fire sergeant	0.36	(0.11–1.17)	0.98	(0.41–2.34)
Fire lieutenant	0.53	(0.22–1.30)	0.56	(0.30–1.03)
**Job duties**				
Others	1.00		1.00	
Fire suppression	0.59	(0.38–0.91)	1.06	(0.78–1.43)
Rescuer	0.64	(0.34–1.21)	0.84	(0.54–1.32)
**Total years of fire service**				
<1	1.00		1.00	
1–10	1.03	(0.21–5.00)	0.29	(0.05–1.82)
10–20	0.36	(0.06–2.35)	0.39	(0.06–2.80)
20–30	0.40	(0.05–3.28)	0.55	(0.07–4.51)
30≤	0.45	(0.05–4.05)	0.62	(0.07–5.25)
**Total years of shift work in the fire service**				
<1	1.00		1.00	
1–10	0.40	(0.11–1.47)	2.37	(0.45–12.37)
10–20	0.69	(0.16–2.88)	2.28	(0.42–12.24)
20≤	0.48	(0.11–2.19)	1.92	(0.35–10.61)
**Monthly number of emergency dispatches in the past year**				
1–10	1.00		1.00	
10–20	0.94	(0.58–1.54)	0.82	(0.59–1.15)
20≤	1.01	(0.57–1.80)	1.12	(0.77–1.62)
**Monthly number of dispatches to incipient-stage or free-burning in the past year**				
None	1.00		1.00	
1–5	1.02	(0.44–2.41)	1.34	(0.69–2.61)
5–10	1.49	(0.59–3.73)	2.06	(1.02–4.17)
10≤	1.36	(0.51–3.67)	2.11	(1.02–4.38)
**Pre-training in SCBA use**				
No	1.00		1.00	
Yes	1.09	(0.60–1.98)	1.02	(0.69–1.50)
**Pre-training in SCBA washing**				
No	1.00		1.00	
Yes	1.86	(1.04–3.31)	1.67	(1.15–2.41)
**Goodness of fit test**				
**Model** **χ^2^**	42.912	(*p* < 0.010)	70.627	(*p* < 0.0001)
**Hosmer and Lemeshow** **χ^2^**	7.594	(*p* < 0.474)	6.417	(*p* < 0.601)
**Nagelkerke *R*^2^**	0.059		0.062	

SCBA: self-contained breathing apparatus, AOR: Adjusted Odds Ratio, CI: Confidence Interval.

## Data Availability

The data that support the findings of this study are available from the corresponding author upon reasonable request.
